# Multi-Stage State Assessment of Breakers Based on TCWGAN-GP and XGBoost Under Insufficient Samples

**DOI:** 10.3390/s26103112

**Published:** 2026-05-14

**Authors:** Lixia Sun, Ling Wang, Jiahao Wang, Zijia Liu

**Affiliations:** School of Electrical and Power Engineering, Hohai University, Nanjing 210098, China

**Keywords:** circuit breaker, condition assessment, data imbalance, TCWGAN-GP, three-stage assessment, XGBoost

## Abstract

The increasing randomness and volatility of renewable energy resources have raised higher demands for circuit breakers. Utilizing monitoring data enables more accurate condition assessment; however, the imbalance between fault and normal samples hampers the performance of machine-learning-based assessment. To address the overfitting and limited diversity of traditional oversampling methods, this paper proposes a Transformer-conditioned CWGAN-GP (TCWGAN-GP) model to generate multi-class fault samples for data augmentation. The generator of the proposed model takes random noise and class labels as input to capture the distribution characteristics of real fault samples. By combining a transformer-based generator to model inter-feature dependencies among 14 monitoring indicators and a WGAN-GP training objective with gradient penalty, the proposed approach improves training stability and synthetic sample quality. Moreover, a three-stage state assessment method based on XGBoost is developed to sequentially assess health status, fault category, and fault severity. Results demonstrate that the proposed method in the paper outperforms conventional data augmentation methods and single-stage classifiers in terms of accuracy, recall, F1-score, and online prediction efficiency. Specifically, the proposed three-stage model achieves an overall assessment accuracy of 93.10%, outperforming the single-stage XGBoost framework. In terms of online efficiency, the initial anomaly detection stage requires only 0.0041 s per sample, which is a substantial reduction compared to the 0.0241 s required by the single-stage model. Furthermore, compared to traditional Random Oversampling (ROS) and SMOTE, the TCWGAN-GP augmentation yields superior evaluation performance on fully balanced datasets, achieving a recall rate of 91.26% and an F1-score of 92.61%. Overall, the proposed TCWGAN-GP and three-stage XGBoost method contributes to addressing data imbalance challenges and improving the accuracy of circuit breaker state assessment.

## 1. Introduction

Circuit breakers are a critical component of power equipment in substations, playing a vital role in controlling and protecting distribution lines. Their condition greatly impacts the operation and maintenance of substations and distribution lines. In recent years, as part of efforts to achieve “carbon peak” and “carbon neutral” targets as soon as possible, distribution networks have been integrated with new energy sources such as photovoltaic and wind power. These new energy sources exhibit fluctuating characteristics, which increase the reliability requirements for circuit breakers. Compared with other substation components such as transformers and protection relays, circuit breakers exhibit a relatively higher failure frequency due to frequent mechanical operations and exposure to harsh environments. According to industry reports [1,2], mechanical failures account for a significant proportion of breaker outages. Failure of circuit breakers may lead to severe consequences, including protection misoperation, cascading faults, and even large-scale power outages. Although redundancy mechanisms such as backup protection systems exist, they cannot fully eliminate the risks associated with breaker failure. Therefore, it is essential to conduct real-time health assessments of circuit breakers to enhance their operation and maintenance [3,4,5].

In the context of the new power system, renewable energy is widely accessed, and the uncertainty faced by the system has increased significantly, making it more important to fully consider the circuit breaker’s own characteristics and environmental meteorological, and other information, and to realize the fusion analysis of multi-source information for a comprehensive and accurate perception of the equipment state. Currently, there are three main methods for assessing the health status of circuit breakers: manual empirical qualitative assessment, statistical analysis, and artificial intelligence. Manual empirical qualitative assessment relies on personal expertise and operational experience, which varies significantly from person to person, making the method highly subjective. Moreover, the complex structure and operation principle of high-voltage circuit breakers limit the practical application of this method [6,7]. Statistical analysis methods use data on circuit breaker operations to determine faults by setting thresholds related to characteristic variables. These methods require high-quality data, and the threshold setting can significantly impact their performance [8]. In contrast, artificial intelligence methods establish a nonlinear mapping relationship between monitoring data and health status by learning from historical circuit breaker operation data through intelligent algorithms, without requiring prior knowledge. In recent years, artificial intelligence techniques have developed rapidly and become the mainstream method for circuit breaker fault diagnosis. Circuit-breaker vibration signal features using wavelet analysis are extracted and evaluated using the mechanical performance employing Support Vector Machine (SVM) techniques [9]. Mechanical fault identification is achieved through a hybrid deep network combining neural networks and long short-term memory (LSTM) units, utilizing convolutional layers to transform raw vibration signals [10]. Similarly, a diagnosis framework for high-voltage circuit breakers is implemented using vibration time-frequency diagrams processed through deep transfer learning [11]. However, most studies only consider a certain characteristic of the circuit breaker during condition assessment and do not consider multidimensional factors such as mechanical characteristics, insulation characteristics, temperature characteristics, and external environmental conditions of the equipment. Additionally, most current studies treat circuit breaker fault diagnosis as a multi-classification problem, without precisely dividing the severity of the same type of fault.

To provide a clearer understanding of existing research, fault diagnosis methods for circuit breakers can be categorized from two complementary perspectives: operational and conceptual. From the operational perspective, methods can be classified as offline, online, or quasi-online approaches, depending on how the diagnostic system is deployed and whether real-time monitoring is supported. From the conceptual perspective, existing approaches can be broadly divided into signal-based, model-based, and data-driven methods. Signal-based methods rely on feature extraction from measured signals and model-based methods utilize physical or mathematical models of the system, while data-driven methods learn patterns directly from historical data using machine learning or deep learning techniques.

In recent years, extensive research has been conducted on intelligent condition assessment methods for power transmission and transformation equipment [12,13,14,15]. From a methodological perspective, data-driven approaches can generally be categorized into traditional pipelines [12,13] and end-to-end pipelines. Traditional pipelines rely on handcrafted feature extraction based on domain knowledge, followed by conventional machine learning algorithms for classification or regression. In contrast, end-to-end pipelines, typically represented by deep learning architectures such as convolutional neural networks (CNNs) [14,15], automatically learn representative features directly from raw monitoring signals.

In the field of condition assessment, traditional machine learning methods remain widely adopted due to their strong interpretability and robustness under limited data conditions. Representative supervised learning algorithms include random forests, decision trees, SVMs, naive Bayes classifiers, and artificial neural networks, while unsupervised learning approaches mainly include clustering algorithms and principal component analysis (PCA). These methods have demonstrated effectiveness in equipment fault diagnosis and state evaluation tasks.

Although the present work focuses on a traditional machine learning framework, end-to-end deep learning approaches capable of directly learning discriminative representations from raw signals are also promising for practical condition assessment. Future work will investigate hybrid deep learning architectures and multimodal end-to-end frameworks to further improve generalization ability and real-world applicability under complex operating conditions [16,17].

According to the State Grid Corporation of China, the health status of distribution equipment is classified into four levels based on the calculated health index: no defect, general defect, significant defect, and emergency defect [18]. Different health indices correspond to different operating states of the distribution equipment parameters. Table 1 presents the health degree of equipment in the distribution network.

The four levels are defined as follows:

(1) No defect (normal): The operating parameters of the distribution equipment are stable, and all operating parameters comply with safety standards, which is displayed in green in the power monitoring system.
(2) General defect: One major operating parameter or multiple operating parameters of the distribution equipment exceed the general defect limit and are gradually approaching the significant defect limit, but have little impact on the normal operation of the equipment. It shows as blue in the power monitoring system.
(3) Significant defect: One major operating parameter or multiple operating parameters of the distribution equipment exceed the significant defect limit and are gradually approaching the emergency defect limit. This has already affected the equipment’s performance and may deteriorate into an emergency defect, but the equipment can still continue to operate. It performs in yellow.
(4) Emergency defect: One or several parameters of the equipment exceed the emergency defect limit or are severely abnormal. The equipment can only operate for a short period or must be shut down immediately. In the power monitoring system, it is shown in red.

In addition, although the current circuit breaker operation data is huge, the circuit breaker fault data is scarce and there is a problem of sample imbalance, which is not conducive to model training for AI-based circuit breaker condition assessment. Currently, traditional fault sample data enhancement methods for this problem contain random oversampling algorithms that enrich the data set by randomly replicating the scarce samples [19]. This method does not lead to information loss, but due to the repetitive nature of the replicated information, overfitting often occurs when the model is trained, which adversely affects the training results. SMOTE has been comprehensively analyzed and applied in recent years [20]. New samples from manual simulations are added to the dataset so that the sample classes of the original data are no longer severely imbalanced. Moreover, several variants of algorithms have been derived based on the SMOTE algorithm [21,22,23,24], such as Borderline-SMOTE [21], SVM-SMOTE [22], adaptive synthetic sampling methods [23], and majority-weighted minority oversampling techniques [24]. These methods generate data based more on difficult samples, thus addressing the problem of overgeneralization of SMOTE. However, these methods do not consider most of the neighboring data when generating samples. They may generate a large amount of duplicate information when there are a few classes of samples. In addition, in some cases, they may not be able to find all the difficult samples among the few samples by neighborhood judgment only.

To address these deficiencies, this paper considers the electrical, mechanical, insulation, temperature, and external environmental characteristics of circuit breakers. And fault sample data of circuit breakers are enriched by a data enhancement method based on the Transformer-conditioned CWGAN-GP (TCWGAN-GP) model, which uses Wasserstein distance to measure the difference between generated and real samples within the GAN framework. Compared with the Jensen–Shannon (JS) or Kullback–Leibler (KL) divergence used in traditional GAN models, the Wasserstein distance has better mathematical properties, is easier to train, and is more stable for generator and discriminator learning. In addition, TCWGAN-GP introduces a gradient penalty mechanism to constrain discriminator gradient size by adding a gradient penalty term to the loss function, thus improving training stability and generated sample quality, which more effectively reduces sample imbalance effects on training compared with traditional data enhancement methods. At the same time, this paper divides the same fault into three degrees: general, critical, and emergency fault, and establishes a three-stage model for health status assessment of circuit breakers, with the first stage judging abnormal operation; the second judging fault type; and the third judging severity. The actual arithmetic results show that the three-stage XGBoost model proposed in this paper has higher evaluation performance and accuracy compared with basic models such as SVM and the single-stage XGBoost model.

## 2. Data Set Construction

This section describes the construction of the dataset used in this study. Relevant features are selected based on the physical characteristics and operational behavior of circuit breakers, ensuring that the data reflect key degradation mechanisms. Then, fault simulation experiments are conducted to generate representative samples under different operating conditions. Therefore, data preprocessing techniques are applied to standardize the dataset and improve its suitability for subsequent model training. The overall process aims to provide a reliable and physically meaningful dataset for condition assessment.

### 2.1. Feature Selection

The main operating characteristics and operating parameters of the circuit breaker are as follows.

(1) Temperature and weather characteristics: Due to the large load currents flowing through the circuit breaker, factors such as contact position misalignment can lead to poor contact, thereby generating severe heat. The temperature characteristics of the circuit breaker can be represented by the contact temperature and the relative temperature difference.
(2) Mechanical and electrical characteristics: The mechanical characteristic parameters of the circuit breaker mainly include: opening and closing time, contact travel, opening and closing speed, average opening and closing speed, maximum opening and closing speed, opening and closing coil current, breaking current, vibration signals, etc.
(3) Insulation characteristics: The monitoring of the insulation characteristics of the equipment’s arc extinguishing chamber mainly involves monitoring the trace moisture content within the SF6 arc extinguishing chamber, the oxygen and SF6 concentration or gas pressure, and the degree of vacuum in the vacuum arc extinguishing chamber.

**Figure 1:**
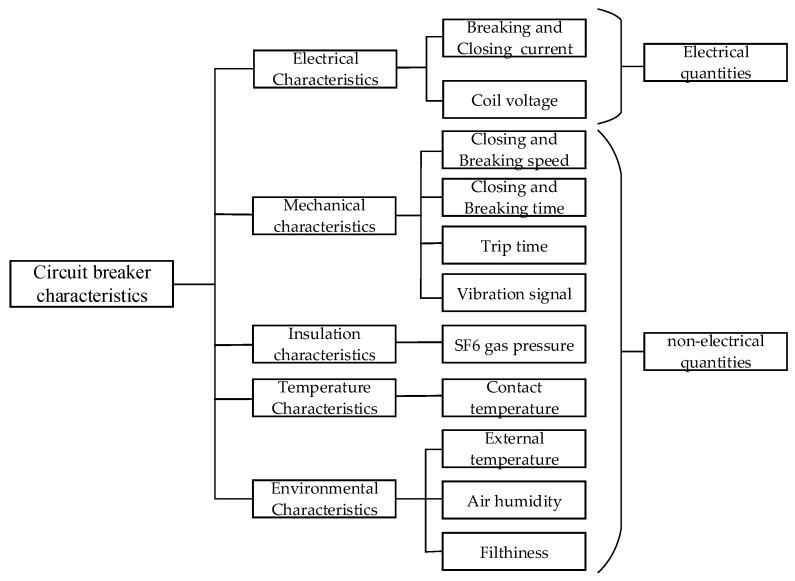
Main operating parameters of distribution circuit breakers.

These main electrical and non-electrical parameters of the circuit breaker are classified into four states according to standard criteria: defect-free, general defect, major defect, and emergency defect. The selected main operating parameters of the circuit breaker and the defect limits for each parameter are shown in Figure 1.

The performance of circuit breakers manifests through various aspects, including electrical characteristics, mechanical characteristics, insulation characteristics, temperature characteristics, and other data. Additionally, the operation of circuit breakers can be influenced by external environmental factors [25,26]. To provide comprehensive insights, this paper incorporates these factors and combines them with findings from our own experiments. Table 2 presents the selected characteristics for this study, encompassing different types of features.

The above characteristic quantities of the circuit breaker are obtained by the following sensor arrangement scheme and data monitoring scheme:

1. Install angular displacement sensors on the side of the main shaft of the circuit breaker to monitor the main shaft turning angle and calculate the contact stroke according to the functional relationship, and determine whether the breaking and closing are in place according to the angular displacement;
2. Arrange pressure sensors on the tie rods of the circuit breaker to deduce some mechanical characteristics of the circuit breaker in combination with the angular displacement sensors;
3. Install acceleration sensors on the operating mechanism to monitor the vibration signal of the circuit breaker;
4. Arrange infrared sensors next to the circuit breaker to monitor the contact temperature;
5. Electrical quantities are measured by current and voltage sensors;
6. Environmental characteristics data are obtained according to the weather station;
7. Filthiness is quantified based on equivalent salt deposit density, which reflects the level of surface contamination.

These features are derived from sensor measurements through physical relationships, ensuring interpretability.

### 2.2. Fault Simulation

The data presented in this paper is obtained from simulated fault tests performed on 10 kV circuit breakers. It is crucial to acknowledge that the lifespan of circuit breakers imposes limitations on the number of fault simulation experiments that can be conducted. In this study, Table 3 furnishes comprehensive details on the fault types, fault simulation modes, and the corresponding sample sizes utilized.

The same fault, depending on its severity, can be divided into general, critical and emergency faults:

1. General fault: The circuit breaker can perform the function as specified; some parts of the performance degrade and risk resistance is reduced, but the degree of impact is small.
2. Critical fault: The circuit breaker can perform the function as specified. However, certain components may experience significant performance degradation, which increases the risk of failure and leads to significant consequences.
3. Emergency fault: The circuit breaker is unable to perform the function as specified; the degree of impact is very high.

The sample numbers of different degrees of faults are shown in Table 4. Considering that different degrees of faults are different for the maintenance plan of the power grid company, the paper therefore divides the severity of the same type of fault. Taking the national grid company as an example, general faults require processing within 6 months; critical faults require processing within 7 days; and emergency faults require processing on the same day. Therefore, it is meaningful to conduct the severity assessment, which can provide fine guidance to the maintenance work arrangement of operations and maintenance personnel.

### 2.3. Data Pre-Processing

The circuit breaker health status assessment based on an artificial intelligence algorithm requires high-quality data, which generally comes from equipment operation and experiments. The equipment that has been put into application has not been installed with sensors, and the feature vectors are not easily collected, so the data are mostly obtained through experiments, but the data obtained from experiments are too idealized, and there is still a gap between the model trained by experimental data and the actual application. In order to further improve the state sensing capability of the circuit breaker equipment, it is necessary to carry out the installation of relevant sensors to realize the real-time assessment of circuit breaker health level.

In future applications, if relevant sensors are installed in the circuit breaker, it would enable the acquisition of equipment operating data, which can be used to train an artificial intelligence-based model for circuit breaker health status assessment. However, it is essential to have an adequate number of fault samples to address the issue of sample imbalance. To overcome the limitations of insufficient samples and imbalanced categories, the TCWGAN-GP model introduced in Section 3 is employed to generate fault samples for circuit breakers, thereby enriching the sample dataset. The health indices are used to label the sample categories, and the functional relationship is expressed as follows:

(1) $$H_{n}=\left\{\begin{array}{l} \begin{array}{ll} 3+\left|\frac{x_{n}-x_{n}^{E}}{x_{n}^{E}}\right| & x_{n}^{E_{\max}}\leq x_{n}\;\mathrm{or}\;x_{n}\leq x_{n}^{E_{\min}}\\ 2+\left|\frac{x_{n}-x_{n}^{I}}{x_{n}^{E}-x_{n}^{I}}\right| & \begin{array}{l} x_{n}^{I_{\max}}\leq x_{n}<x_{n}^{E_{\max}}\;\mathrm{or}\\ x_{n}^{E_{\min}}<x_{n}\leq x_{n}^{I_{\min}} \end{array}\\ 1+\left|\frac{x_{n}-x_{n}^{G}}{x_{n}^{I}-x_{n}^{G}}\right| & \begin{array}{l} x_{n}^{G_{\max}}\leq x_{n}<x_{n}^{I_{\max}}\;\mathrm{or}\\ x_{n}^{I_{\min}}<x_{n}\leq x_{n}^{G_{\min}} \end{array}\\ \left|\frac{x_{n}}{x_{n}^{G_{\max}}-x_{n}^{G_{\min}}}\right| & x_{n}^{G_{\min}}\leq x_{n}<x_{n}^{G_{\max}} \end{array}\\ n=1,\;2,\;\ldots ,\;S \end{array}\right.$$

(2) $$H=\max{\left\{H\right.}_{1},H_{2},\ldots \;,H_{n},\ldots \;,\left.H_{S}\right\}$$

where *S* is the total number of fault categories, and *H_n_* is the health level of the breaker corresponding to fault number *n*. When *H_n_* is [0, 1), the breaker parameters are fault-free; when *H_n_* is [1, 2), the breaker parameters are in a general fault state; when *H_n_* is [2, 3), the equipment parameters are in a critical fault state; and when *H_n_* is [3, 4), the equipment parameters are in an emergency fault state. *x_n_^Gmax^*, *x_n_^Gmin^*, *x_n_^Imax^*, *x_n_^Imin^*, *x_n_^Emax^*, *x_n_^Emin^* are the upper and lower limits of general, critical and emergency faults, respectively. In this paper, only a single fault of the circuit breaker is considered, and the overall health level of the circuit breaker *H* is the health index of the device according to the health index of the worst parameter.

It should be noted that the degradation states in this study are generated through controlled fault simulation experiments, which may not fully capture the complexity of natural aging processes in real-world conditions. However, the simulation scenarios are designed based on known physical degradation mechanisms of circuit breakers, ensuring that the generated data reflect realistic variations in key measurable quantities.

In addition, the selected features are directly derived from physical signals such as current and vibration, which are closely related to actual operating conditions. Therefore, although the data are obtained under laboratory conditions, they preserve essential characteristics of real degradation behaviors.

## 3. Proposed Method for TCWGAN-GP-Based Data Augmentation and Three-Stage XGBoost State Assessment

This section presents the proposed methodology for circuit breaker condition assessment. It first introduces the TCWGAN-GP model for data augmentation, which is designed to address the issue of insufficient and imbalanced fault samples. Then, a three-stage XGBoost-based framework is developed to achieve progressive assessment, including abnormality detection, fault type identification, and severity evaluation. The integration of data augmentation and hierarchical classification aims to improve both the accuracy and robustness of the overall system.

### 3.1. Overview of the Proposed Framework

**Figure 2:**
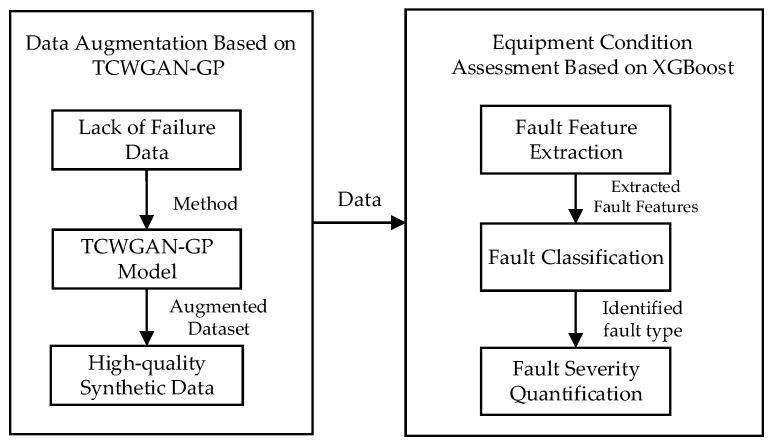
Overall framework of data augmentation based on TCWGAN-GP and three-stage XGBoost state evaluation method.

This paper proposes a three-stage state assessment framework for medium-voltage circuit breakers under insufficient and imbalanced fault samples by combining Transformer-conditioned CWGAN-GP (TCWGAN-GP) based data augmentation with XGBoost classifiers. Figure 2 shows the overall framework of data augmentation based on TCWGAN-GP and the three-stage XGBoost state evaluation method.

First, multi-source condition-monitoring features (mechanical, electrical, insulation, temperature, and environmental variables) are organized into 14-dimensional feature vectors and standardized using the z-score method.

To alleviate fault-sample scarcity and imbalance, TCWGAN-GP is trained to generate class-conditioned synthetic samples. The model adopts the Wasserstein distance to measure distribution discrepancy and introduces a gradient penalty term to enforce the 1-Lipschitz constraint, improving training stability and sample quality. In addition, a transformer-based generator is used to model feature dependencies, and an attention-enhanced critic with an auxiliary classification head is employed to strengthen conditional learning.

In our implementation, the noise dimension is set to 14, batch size is 32, the gradient penalty coefficient is 10, and the Adam optimizer is used with an initial learning rate of 0.0005 (*β*_1_ = 0.5, *β*_2_ = 0.9). The model is trained for 5000 iterations.

After augmentation, a three-stage XGBoost-based assessment strategy is built to achieve refined state evaluation: Stage 1 detects whether the breaker is abnormal; Stage 2 identifies the fault category; Stage 3 evaluates fault severity for the identified fault type using a dedicated model per category.

Model hyperparameters are selected via grid search. The classification performance is evaluated using accuracy (A) and missed-alarm rate (MA) in Stage 1, and accuracy in Stages 2 and 3.

### 3.2. Sample Enhancement Method Based on Transformer-Conditioned CWGAN-GP Model

In the application of artificial intelligence algorithms for the condition assessment of circuit breakers, high-quality and sufficient data is a critical prerequisite for training reliable models. However, in practical operations and simulation experiments, normal operation data is abundant, whereas fault samples are often extremely scarce. This leads to a severe problem of sample category imbalance, which significantly hinders the learning performance of the models. Although traditional oversampling methods (such as random oversampling or the SMOTE algorithm) can enrich the dataset to some extent, they are prone to causing model overfitting or generating a large amount of redundant and invalid sample information. To overcome these limitations and generate high-quality fault samples, this section introduces a data augmentation method based on the Transformer-conditioned CWGAN-GP (TCWGAN-GP) model. This method combines the powerful global feature dependency modeling capability of the Transformer with the superior distribution fitting stability of WGAN-GP, aiming to provide a more balanced data foundation with realistic physical distribution characteristics for the subsequent condition assessment.

#### 3.2.1. Principle of GAN Model and Its Variant Networks

Generative Adversarial Networks (GANs) have been widely researched and applied in various fields [27]. A GAN contains two models: a generator *G* and a discriminator *D*. The generator maps a noise vector to the data space and aims to produce samples that resemble real data, while the discriminator aims to distinguish generated samples from real ones. During adversarial training, *G* and *D* are optimized alternately with competing objectives, and they gradually improve their generation and discrimination capabilities until reaching a stable equilibrium.

When the GAN model is used to generate circuit breaker fault samples, assume that n sets of circuit breaker fault samples are currently obtained, and the circuit breaker fault samples are grouped in order as ***x*** = {*x*_1_, *x*_2_, …, *x_n_*}, where *x_n_* represents the state feature vector of the *n*th set of circuit breaker samples. Assuming that the probability distribution of the circuit breaker fault samples ***x*** is denoted as *P_r_*, and the noise vector ***z*** obeys the Gaussian distribution *P_g_*, the GAN model makes the generated samples mapped by Gaussian noise ***z*** have the same distribution law as the actual fault samples by establishing the mapping relationship from *P_g_* to *P_r_*.

In the training process, the noise vector ***z*** is used as the input of the generator, and generated data *G*(***z***) with the same dimension as the actual fault sample ***x*** is obtained after forward propagation. Then, the real fault sample ***x*** and the generated data *G*(***z***) are jointly fed into the discriminator to determine whether the input comes from the real data distribution. To quantify the discrepancy between the distributions of *G*(***z***) and ***x***, the GAN training loss is defined as the following:

(3) $$L_{G}=E_{ɀ\sim Pg}[\ln(1-D(G(ɀ)))]\;$$

(4) $$L_{D}=-E_{x\sim P_{r}}[\ln D(x)]-E_{ɀ\sim P_{g}}[\ln(1-D(G(ɀ)))]$$

where *E*(∙) denotes the computational expectation; *G*(***z***) denotes the artificial sample synthesized by the generator; and *D*(∙) denotes the output of the discriminator.

Based on these components, the generator and discriminator undergo iterative optimization to enhance their respective generation and discrimination capabilities. Through this iterative process, they gradually reach a state of equilibrium, where the discriminator struggles to distinguish between the “fake data” generated by the generator and the real faulty samples. The final objective function of GAN model training can be obtained by combining Equations (3) and (4), which is shown in the following Equation (5):

(5) $$\min_{G}\max_{D}V(G,D)=E_{x\sim P_{r}}[\ln D(x)]+E_{ɀ\sim P_{z}}[\ln(1-D(G(ɀ)))]$$

**Figure 3:**
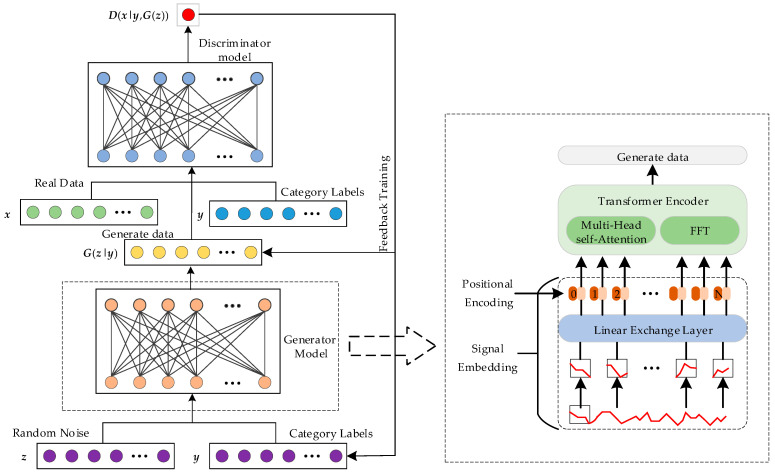
Structure of TCWGAN-GP.

The traditional GAN model mainly contains two problems: (1) the loss function of the generator faces the problem of gradient disappearance, and the root of this problem is mainly rooted in the unreasonable distance measure (JS divergence, KL divergence) of the equivalent optimization during the training process; (2) the pattern collapse problem, as the generator and discriminator will not be able to converge simultaneously during the training process, resulting in the failure of the model training, resulting in generated samples whose quality does not meet the requirements [28]. In order to solve the above two problems, the improved GAN model—the TCWGAN-GP model, which is used in this paper—is optimized for the above problems, and its basic model architecture is shown in Figure 3.

To avoid the above problem, the CWGAN-GP model replaces the JS scatter in the traditional model with the Wasserstein distance when measuring the gap between the generated sample distribution and the actual sample distribution, which is defined as shown in Equation (6). The smaller the value of *W*(*P_r_*, *P_g_*), the more similar the distribution between *P_r_* and *P_g_*.

(6) $$W\left(P_{r},P_{g}\right)=\underset{\gamma \sim \Pi \left(P_{r},P_{g}\right)}{\inf}E_{(x,y)\sim \gamma }[\|x-y\|]$$

where *P_r_*, *P_g_* are the distributions of real and generated data; Π(*P_r_*, *P_g_*) is the joint probability distribution of *P_r_* and *P_g_* combined; ***x*** represents a real sample drawn from the true data distribution *P_r_*, and ***y*** denotes the corresponding conditional label used in the conditional generative framework.

Since the lower bound taken for this expectation in the joint probability distribution cannot be solved directly, the pairwise form of the Wasserstein distance is used, as shown in Equation (7). Meanwhile, by substituting the label ***y*** of the data also as the input of the discriminator into Equation (6), the representation of the Wasserstein distance considering the category label y can be obtained, as shown in Equation (8).

(7) $$W\left(P_{r},P_{g}\right)=\underset{\|D{\|}_{L\leq 1}}{\sup}E_{x\sim P_{r}}[D(x)]-E_{ɀ\sim P_{z}}[D(G(ɀ))]$$

(8) $$W\left(P_{r},P_{g}\right)=\underset{\|D{\|}_{L\leq 1}}{\sup}E_{x\sim P_{r}}[D(x)]-E_{ɀ\sim P_{z}}[D(G(ɀ))]$$

where ||*D*||*_L_*_≤1_ indicates that the discriminator *D* needs to satisfy the Lipschitz continuity condition and *L* ≤ 1; ***z*** denotes the input noise vector sampled from a prior distribution.

In this paper, with the aim of optimizing the loss function to be more efficient and stable, a gradient penalty term is added to the discriminator. So, it satisfies the Lipschitz continuity condition, which restricts the function optimization by providing an upper bound to the optimization problem. Its form is shown in the following Equation (9):

(9) $${\left.GP\right|}_{\hat{x}}=\lambda E_{\hat{x}\sim P_{\hat{x}}}[{\left(||\nabla_{\hat{x}}D(\hat{x})|{|}_{p}-1\right)}^{2}]$$

where ||∙||*_p_* denotes the p-parameter; λ denotes the gradient penalty coefficient; $\hat{x}$ is obtained by random sampling between the actual fault sample ***x*** and the generated sample *G(**z**)*. The expression of random sampling is $\hat{x}$ = *ε**x*** + (1 − *ε*)*G*(***z***), where parameter *ε* satisfies a uniform distribution in the range [0, 1].

Substituting the gradient penalty term *GP* of the above equation into the discriminator loss function of the conventional GAN model, the loss functions are obtained in the generative network and discriminator network of the CWGAN-GP model as shown in Equations (10) and (11), respectively.

(10) $$L_{G}=-E_{z\sim P_{z}}[D(G(ɀ|y)|y)]$$

(11) $$L_{D}=E_{ɀ\sim P_{z}}[D(G(ɀ|y)|y)]-E_{x\sim P_{r}}[D(x|y)]+\lambda E_{\hat{x}\sim P_{\hat{x}}}[{\left(||\nabla_{\hat{x}}D(\hat{x})|{|}_{p}-1\right)}^{2}]$$

In the proposed TCWGAN-GP, the generator is implemented using a Vision Transformer (ViT)-based architecture. Specifically, the random noise vector ***z*** and the conditional label ***y*** are first fused and projected into a latent feature representation, which is then reshaped into a sequence of embedded tokens and fed into the Transformer encoder for global dependency modeling. In this way, the conditional generation process *G*(***z***, ***y***) is explicitly realized through the ViT-based generator.

#### 3.2.2. Architecture of the Vision Transformer Encoder

**Figure 4:**
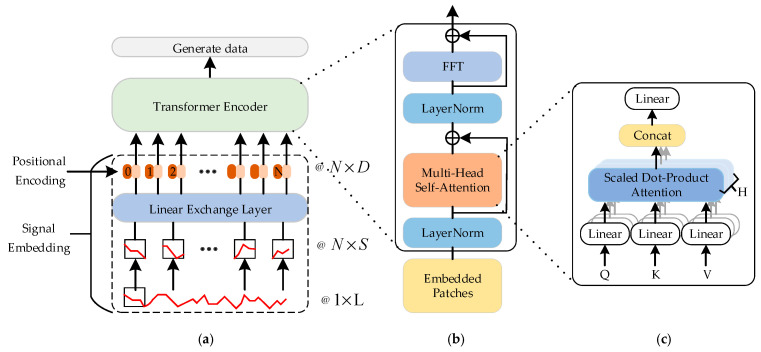
Architecture of the Vision Transformer encoder. (**a**) Overall structure. (**b**) Transformer encoder. (**c**) Multi-head self-attention mechanism.

In the proposed TCWGAN-GP, the generator is implemented using a Vision Transformer-based architecture. Specifically, the random noise vector ***z*** and the conditional label ***y*** are first fused and projected into a latent feature space, and then transformed into a sequence of input tokens for the Transformer encoder, thereby realizing the conditional generation process *G*(***z***, ***y***). Figure 4 presents the framework of the architecture of the Vision Transformer encoder.

Figure 4a shows the overall structure, which includes signal embedding, positional encoding, a transformer encoder and generating data. The encoder architecture is illustrated in Figure 4b. The transformer encoder is typically composed of a stack of multiple Transformer blocks, each of which consists of a multi-head self-attention (MHSA) layer and a feed-forward network (FFN). These two sublayers are connected through residual connections (RC) and followed by layer normalization (LN), which accelerates model convergence and enhances robustness. The input sequence is first mapped by an embedding layer (EL) into vectors of a specified dimension. It is then partitioned, under the Vision Transformer (ViT) architecture, into (N) patch blocks of size (S), which are subsequently flattened and concatenated. Thereafter, according to Equations (12) and (13), positional encoding (PE) is applied to inject positional information into each input patch, thereby preserving the sequential order of the original sequence.

(12) $$PE_{\left(pos,2i\right)}=\sin\left(pos/n^{2i/d_{model}}\right)$$

(13) $$PE_{\left(pos,2i+1\right)}=\cos\left(pos/n^{2i/d_{model}}\right)$$

where *pos* denotes the element position, *i* is the dimension index, and *d_model_* represents the embedding dimension of the sequence patches.

A sine function is used for even dimensions, whereas a cosine function is used for odd dimensions. Through the alternating use of these functions, the relative positional information among inputs can be captured, thereby assigning each element a dedicated positional encoding vector.

Figure 4c presents the multi-head self-attention mechanism. In the MHSA layer, the input is linearly transformed *H* times and projected into multiple subspaces, where the corresponding attention weights are computed to capture different fault-related features in the input sequence. Finally, the outputs of all attention heads are concatenated and fused through a linear transformation, with dimensionality reduction performed to obtain a single integrated representation vector, as specifically described in Equations (14) and (15). In this way, the relationships between each patch and all other patches can be precisely modeled, enabling the network to learn long-range dependencies.

(14) $$Multihead\left(Q,K,V\right)=Concat\left(head_{1},\cdot \cdot \cdot ,head_{i}\right)W^{0}$$

where *Q*, *K*, and *V* denote the query, key, and value matrices, respectively.

The computation process of each attention head is given by:

(15) $$head_{i}=Attention\left(QW_{i}^{Q},KW_{i}^{K},VW_{i}^{V}\right)$$

Finally, the extracted features are further subjected to nonlinear transformation by the FFN, which compensates for the limitations of MHSA in local feature modeling. In this way, the two components complement each other and jointly enhance the feature representation capability of the Transformer block. By stacking multiple Transformer blocks, the depth of the encoder can be increased, enabling the progressive extraction of deeper features from the input and thereby improving the representational power of the model.

The Transformer-based generator improves feature dependency modeling by leveraging the self-attention mechanism to capture global relationships among input features. Unlike traditional MLP-based generators, which primarily model local or pairwise interactions through fixed network structures, the Transformer dynamically learns the relevance between all feature dimensions. In this study, the input consists of 14 heterogeneous monitoring indicators, such as mechanical, electrical, insulation, temperature, and environmental features, which exhibit complex interdependencies driven by underlying physical processes. Variations in mechanical behavior may simultaneously affect vibration signals, operating time, and electrical responses. The self-attention mechanism enables the model to assign adaptive weights to these cross-feature relationships, thereby learning high-order dependencies that are difficult to capture with conventional architectures. As a result, the Transformer-based generator can better approximate the joint distribution of real fault data, leading to more realistic and diverse synthetic samples.

### 3.3. Three-Stage Condition Assessment Based on XGBoost

Following the data augmentation process utilizing the TCWGAN-GP model, the sample imbalance issue is effectively alleviated, creating favorable conditions for constructing a highly accurate condition assessment model. Currently, most existing studies on circuit breaker fault diagnosis tend to employ a single multi-class model framework. However, requiring a model to simultaneously distinguish all health states and degradation levels significantly increases the complexity of decision boundaries. Particularly when the feature differences between adjacent degradation states overlap, the performance of a single classifier often falls short of practical requirements. Taking into account the varying processing timeframes required for faults of different severities (such as general, critical, and emergency faults) in actual power grid operation and maintenance, this section proposes a three-stage condition assessment framework based on the XGBoost algorithm. By adopting a hierarchical and progressive strategy, this framework logically decomposes the complex classification task into three sequential stages: abnormality detection, fault category identification, and fault severity evaluation. This approach effectively reduces inter-class complexity and better aligns with practical on-site maintenance decision-making procedures.

#### 3.3.1. Three-Stage Model Construction

**Figure 5:**
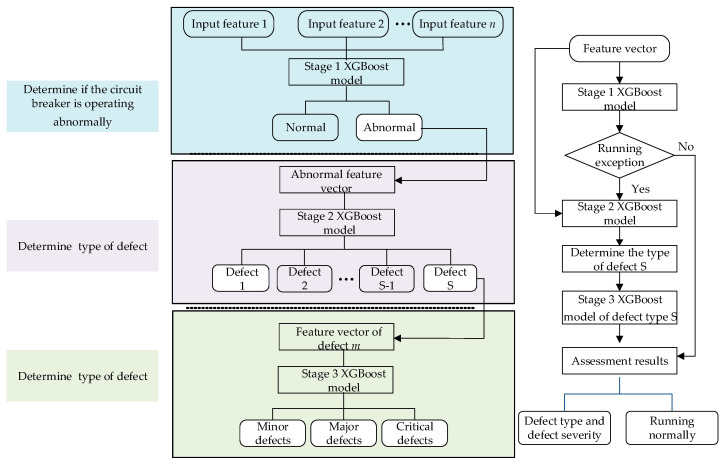
Framework of the three-stage XGBoost model.

The three-stage model constructed in this paper is shown in Figure 5. The first-stage model determines whether the circuit breaker is abnormal based on the feature vector. If an abnormality is detected, the second-stage model identifies the fault category. After the fault category is determined, the third-stage model determines the severity of the fault, and each fault category is modeled separately.

The XGBoost algorithm is used in each stage, and its principle is described in detail in Section 3.3.2. A three-stage design is adopted instead of a single multi-class classifier because it reduces class complexity, alleviates imbalance propagation, and better matches practical maintenance decision-making procedures. The superior performance of the proposed three-stage framework compared to a single multi-class XGBoost model can be attributed to its hierarchical decomposition of the classification task. In a single multi-class setting, the model is required to simultaneously distinguish between all degradation levels, which increases the complexity of the decision boundaries, especially when the differences between adjacent states are subtle and overlapping. In contrast, the three-stage framework decomposes the problem into a sequence of simpler binary or low-complexity classification tasks. Each stage focuses on distinguishing a specific level of degradation severity, allowing the model to learn more discriminative features for that particular decision. This staged learning process reduces intra-class variability and alleviates the confusion between neighboring degradation states. Moreover, the intermediate stage of the three-stage method can output the fault type and sort the faults accordingly.

#### 3.3.2. Principle of XGBoost Algorithm

The XGBoost algorithm was developed by Tianqi Chen et al. The XGBoost algorithm has demonstrated superior performance in circuit breaker condition assessment [29]. Assuming that the dataset D{(*x_i_*, *y_i_*):*i* = 1, 2, …, *N*} has N samples, each with M-dimensional features, *x_i_* denotes the input feature vector of the *i*th sample, and *y_i_* is the corresponding label. The predicted output of the integrated model of K trees can be defined as:

(16) $${\hat{y}}_{i}=\sum_{k=1}^{K}f_{k}(x_{i}),f_{k}\in F$$

where *F* is the set of all regression trees, *F =* {*f*(*x*) *= w_q_*(*x*)}, *q*: *R*^m^⟶*T*, *ω* ∈ *R^T^*, each function *f_k_*(*x*) corresponds to a tree with structure vector *q* and leaf weights *ω*, *T* is the number of leaf nodes, and *f*(*x*) is optimized by the following objective function:

(17) $$L=\sum_{i}l({\hat{y}}_{i},y_{i})+\sum_{k}\Omega (f_{k})$$

where *l* is the error function, which reflects the distance between the predicted and target values; Ω(*f_k_*) is the regularization term of the target function, which prevents overfitting of the model. It is expressed as:

(18) $$\Omega (f_{k})=\gamma T+\frac{1}{2}\lambda {\left\|\omega \right\|}^{2}=\gamma T+\frac{1}{2}\lambda \sum_{j=1}^{T}\omega_{j}^{2}$$

where *λ* and *γ* are penalty coefficients. The above objective function is difficult to optimize in the traditional way. Gradient boosting tree is an improved algorithm of boosting tree, and the model is trained by the forward addition iteration method, and the objective function of the *t*th iteration can be depicted as:

(19) $$\Psi^{\left(t\right)}=\sum_{i=1}^{n}l(y_{i},{\hat{y}}_{i}^{t-1}+f_{t}(x_{i}))+\Omega (f_{t})$$

A quadratic Taylor formula expansion of the above equation is performed, and the objective function for the *t*th iteration is simplified and can be described as:

(20) $$\Psi^{\left(t\right)}=\sum_{i=1}^{n}[l(y_{i},{\hat{y}}_{i}^{t-1})+g_{i}f_{t}(x_{i})+\frac{1}{2}h_{i}f_{t}^{2}(x_{i})]+\Omega (f_{t})$$

where *g_i_* and *hi* are the first and second-order derivatives of the loss function.

Respectively, the objective function can be rewritten as:

(21) $$\Psi^{\left(t\right)}=\sum_{j=1}^{T}\left[(\sum_{i\in I_{j}}g_{i})\omega_{j}+\frac{1}{2}(\sum_{i\in I_{j}}h_{i}+\lambda )\omega_{j}^{2}\right]+\gamma T$$

where *I_j_* = {*i*|*q*(*x_i_*) = *j*} is the set of leaves *j*.

When the structure of tree *q* is fixed, the optimal weights of leaf *j* can be described as:

(22) $$\omega_{j}^{*}=-\frac{\sum_{i\in I_{j}}g_{i}}{\sum_{i\in I_{j}}h_{i}+\lambda }$$

Bringing (22) into the objective function (21), the optimal objective value is calculated as:

(23) $$\Psi (q)=-\frac{1}{2}\sum_{j=1}^{T}\frac{{\left(\sum_{i\in I_{j}}g_{i}\right)}^{2}}{\sum_{i\in I_{j}}h_{i}+\lambda }+\gamma T$$

Since traversing all tree structures incurs a huge computational cost, tree partitioning can be performed by the greedy algorithm, which starts with a single leaf, and the gain for a specific partitioning scheme can be expressed as:

(24) $$Gain=\frac{1}{2}[\frac{{\left(\sum_{i\in I_{L}}g_{i}\right)}^{2}}{\sum_{i\in I_{L}}h_{i}+\lambda }+\frac{{\left(\sum_{i\in I_{R}}g_{i}\right)}^{2}}{\sum_{i\in I_{R}}h_{i}+\lambda }+\frac{{\left(\sum_{i\in I}g_{i}\right)}^{2}}{\sum_{i\in I}h_{i}+\lambda }]-\gamma$$

where *I_L_* and *I_R_* are the sample sets of the left and right nodes after splitting.

#### 3.3.3. Evaluation Indicators

The parameter optimization of the XGBoost algorithm needs to be combined with the evaluation metrics and the practical application of circuit breakers. Considering the different target tasks of each stage of the three-stage model, the accuracy and the missed-alarm rate are selected as the model evaluation indexes for the first-stage model, and the accuracy is selected as the model evaluation indexes for the second- and third-stage models. The accuracy can reflect the comprehensive performance of the model, and missed alarms have a significant impact on grid operation; therefore, the missed-alarm rate is used as an evaluation metric, and the accuracy rate A and the missed-alarm rate are defined as:

(25) $$A=\frac{\sum_{i=1}^{S}{\mathrm{TP}}_{i}}{\sum_{i=1}^{S}\left({\mathrm{TP}}_{i}+{\mathrm{FP}}_{i}\right)}\times 100\%$$

(26) $$\mathrm{MA}=\frac{\mathrm{FN}}{\mathrm{TP}+\mathrm{FN}}\times 100\%$$

where *S* is the number of categories; TP_*i*_ is the number of samples of the *t*th category that are correctly classified; FP_*i*_ is the number of samples that are incorrectly predicted as the *t*th category. FN is the number of samples whose originally abnormal labels are judged as normal labels; TP is the number of samples whose originally abnormal labels are judged as abnormal labels. For imbalanced multi-class tasks (Stage 2 and Stage 3), Macro-F1 and Macro-Recall are also reported to reflect per-class performance.

**Figure 6:**
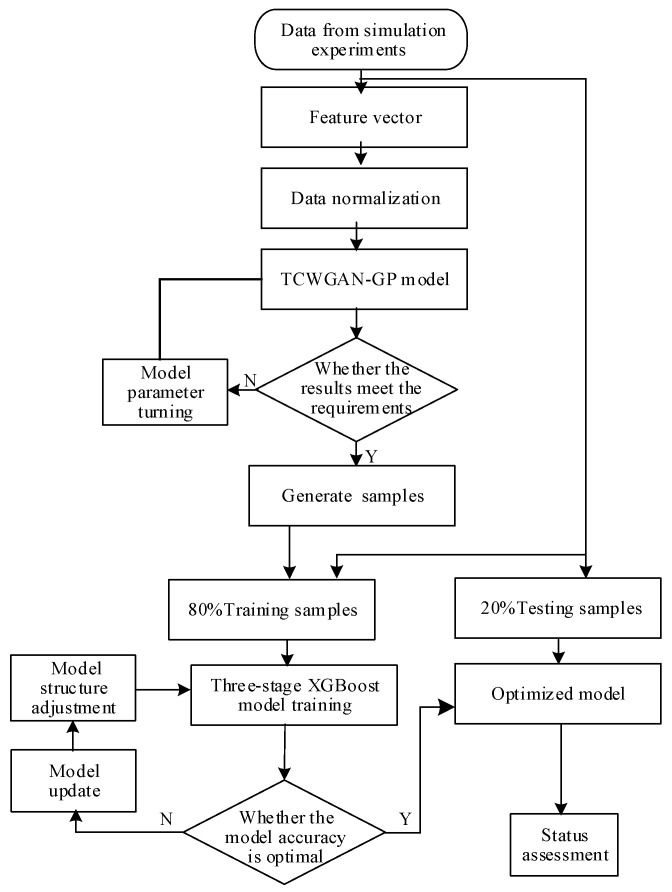
Model training and status assessment flow chart.

The XGBoost algorithm is used to evaluate the model at all stages of the model, and the algorithm trains the prediction process as shown in Figure 6, with the following steps:

1. Data pre-processing: In this paper, the z-score method is used to normalize the data, and the dataset is divided into a training set and a held-out test set (80%/20%). Z-score statistics are computed on the training set and then applied to the test set to avoid data leakage.
2. XGBoost algorithm parameter optimization: In this paper, the optimal parameters are mainly selected by the grid search method. The important hyperparameters of the XGBoost algorithm are decision tree, maximum depth of tree, learning rate, etc. In this paper, the decision tree is selected from the set {100, 200, …, 1000}; the maximum tree depth is selected from {3, 4, …, 10}; the learning rate is selected from {0.1, 0.05, 0.01, 0.005, 0.001}.
3. Model training: Grid search is conducted using 10-fold cross-validation on the training set to select hyperparameters, and the final model is trained on the full training set and evaluated on the held-out test set.

## 4. Algorithm Verification

This section evaluates the effectiveness of the proposed method. It first analyzes the performance of the three-stage XGBoost model in comparison with other baseline approaches. Then, the computational efficiency of the model is assessed in terms of training and prediction time. Finally, the effectiveness of the TCWGAN-GP-based data augmentation method is validated under different sample conditions. These experiments collectively demonstrate the advantages of the proposed framework.

### 4.1. Three-Stage XGBoost Model Identification Performance Evaluation

The PC configuration used in this paper is: Intel(R) Core i7-12700CPU@2.7GHz, the graphics card used is a mobile NVIDIA RTX3060, and the running memory of the computer is 16.00 GB. The TCWGAN-GP model and XGBoost model in this paper are built based on TensorFlow and Keras frameworks.

In this paper, 720 sets of normal samples and 480 sets of abnormal samples (8 fault types, 60 samples each) were obtained through circuit breaker simulation experiments. Data enhancement is performed by TCWGAN-GP to generate 360 sets of normal samples and 600 sets of abnormal samples. For the abnormal class, the generated samples are produced in a class-balanced manner across fault types, that is, 75 sets per fault type, and 25 sets per severity level, to support Stage 2 and Stage 3 tasks. The dataset is divided into training and testing sets in a ratio of 4:1, with a total of 2160 sets of samples. To avoid artificially inflated accuracy, 1728 sets are used for training, which come from data enhancement and simulation experiments, and the remaining 432 sets are utilized for testing, which come from simulation experiments.

The three-stage XGBoost model is compared with the three-stage SVM model, the gradient boosting decision tree (GBDT) model, the stacked auto encoder (SAE) model and TabNet. And the statistical results are shown in Table 5 and Table 6.

As can be seen from Table 5, the XGBoost algorithm outperforms the SVM, GBDT, SAE, and TabNet models, with overall accuracies (A/%) of 4.6%, 4.3%, 2.6%, and 1.9% higher, respectively; and the indicators of missed alarms (MA/%) in the first stage are 2.1%, 1.3%, 0.9% and 0.5% lower, respectively.

TabNet, as a deep learning model designed for tabular data, also demonstrates competitive performance, achieving high accuracy across all stages. However, its overall performance is still slightly inferior to that of the proposed XGBoost model. This may be attributed to the relatively small dataset size, where tree-based models tend to exhibit better robustness and generalization ability compared to deep learning approaches.

For a comparison between the single-stage and three-stage framework model evaluation approaches, performance statistics of XGBoost, SVM, GBDT, SAE and TabNet models are shown in Table 6. From the results shown in Table 5 and Table 6, it can be seen that the overall performance of the three-stage model is significantly better than that of the single-stage framework model. The accuracy rates of the three-stage XGBoost, SVM, GBDT, SAE, and TabNet models are 1.5%, 1.3%, 1.3%, 1.0% and 0.8% higher than those of the single-stage framework models XGBoost_Single, SVM_ Single, GBDT_Single, SAE_Single and TabNet_Single, respectively; and the missed alarm rates are 2.3%, 2.0%, 2.3%, 2.4% and 2.0% lower, respectively.

**Figure 7:**
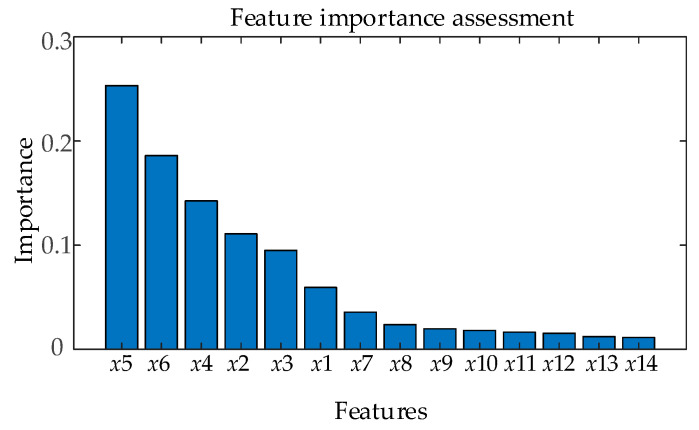
Single-stage XGBoost model feature importance evaluation.

The single-stage XGBoost model can evaluate the importance of each feature, and the feature importance is displayed in Figure 7. As shown in Figure 7, the feature importance assessment results of the single-stage XGBoost model indicate that the mechanical travel time and vibration signal of the circuit breaker are significantly better than the importance of dirtiness and air humidity environ-mental features. These results are consistent with the operational experience of circuit breaker equipment operation and maintenance personnel. However, the single-stage XGBoost assessment model cannot conduct targeted assessments for different fault types.

**Figure 8:**
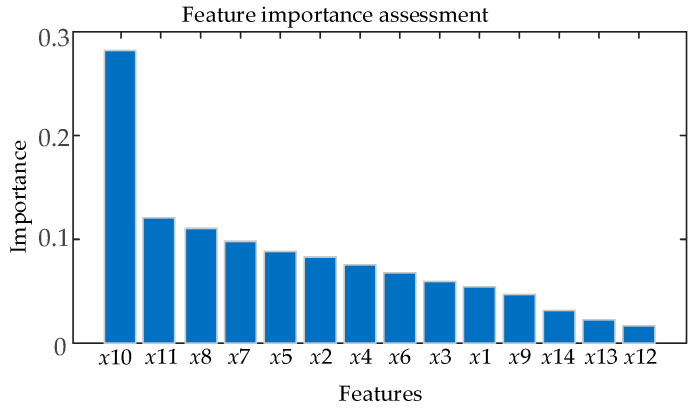
Three-stage SF6 gas pressure fault degree XGBoost model feature importance evaluation.

Taking the three-stage XGBoost evaluation model as an example, the importance ranking of the features of the model in the evaluation of the SF6 gas pressure deficiency fault is shown in Figure 8. As can be seen from the figure, the three-stage XGBoost model of SF6 gas pressure is the most important feature in the evaluation process for this type of fault, which is in line with the operational experience of the circuit breaker equipment operation and maintenance personnel and shows the validity and reasonableness of the three-stage model.

### 4.2. Time Cost Performance Evaluation

The anomaly identification of circuit breakers in substations needs to be evaluated online, so it is hoped that the model identification speed is as fast as possible and the development time is as short as possible. In the paper, the prediction time and training time of the three-stage XGBoost model are compared with the single-stage models of XGBoost_Single, SVM_Single, GBDT_Single, and SAE_Single, as shown in Table 7 and Table 8.

**Figure 9:**
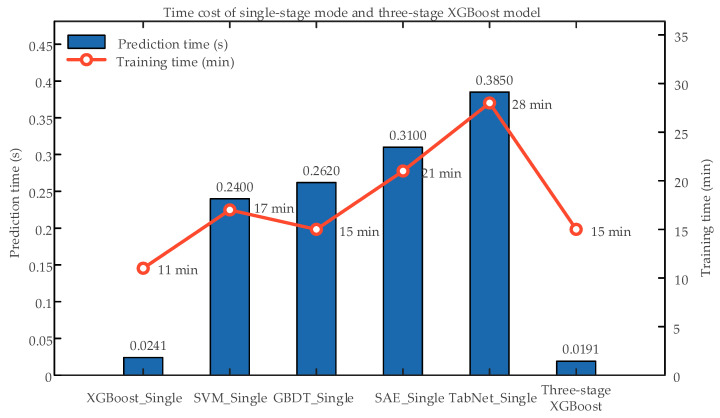
Time cost of single-stage mode and three-stage XGBoost model.

From Table 7 and Table 8 and Figure 9, the three-stage XGBoost model achieves a shorter prediction time than the single-stage models while maintaining a comparable training cost. In practical substations, circuit breakers operate normally for most of the time; therefore, state assessment often terminates at Stage 1. The Stage 1 XGBoost model requires only 0.0041 s per sample, which is substantially lower than the 0.0241 s required by the single-stage XGBoost model. This reduction decreases the overall computational burden and deployment cost for large-scale online monitoring.

### 4.3. Evaluation of TCWGAN-GP-Based Data Enhancement Effect

Artificial intelligence-based circuit breaker health assessment requires high-quality data, and the data obtained by simulating faults in circuit breakers is limited and costly. To simulate the real operation, the number of normal samples is much more than the number of fault samples, and there is an imbalance of sample categories. In this paper, we test the data enhancement effect by generating fault samples and continuously changing the ratio and number of normal and fault samples through TCWGAN-GP-based data enhancement method.

**Figure 10:**
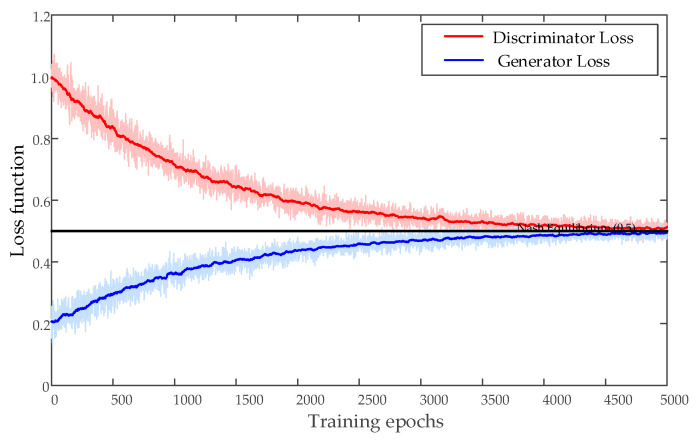
Changes in generator and discriminator losses.

In this paper, the data enhancement model adopts the proposed TCWGAN-GP architecture. The generator is a transformer-based network that models dependencies among the 14 monitoring indicators, while the critic incorporates a feature-level attention module and an auxiliary classification head to strengthen conditional learning. ReLU/LeakyReLU activations are used in hidden layers; since the inputs are standardized, the generator output uses a linear activation to produce 14-dimensional synthetic feature vectors. The critic outputs an unconstrained real-valued score through a linear layer (no sigmoid), consistent with the WGAN-GP formulation. The noise dimension is set to 14, the batch_size is 32, and the gradient penalty coefficient is 10. The model is trained using the Adam optimizer with an initial learning rate of 0.0005 (*β*_1_ = 0.5, *β*_2_ = 0.9) for 5000 iterations. Figure 10 illustrates the training curves; after about 4000 iterations, the critic/generator objectives and the gradient penalty term become stable, indicating that the adversarial training has converged to a steady state and the generated samples are close to the real fault samples.

**Figure 11:**
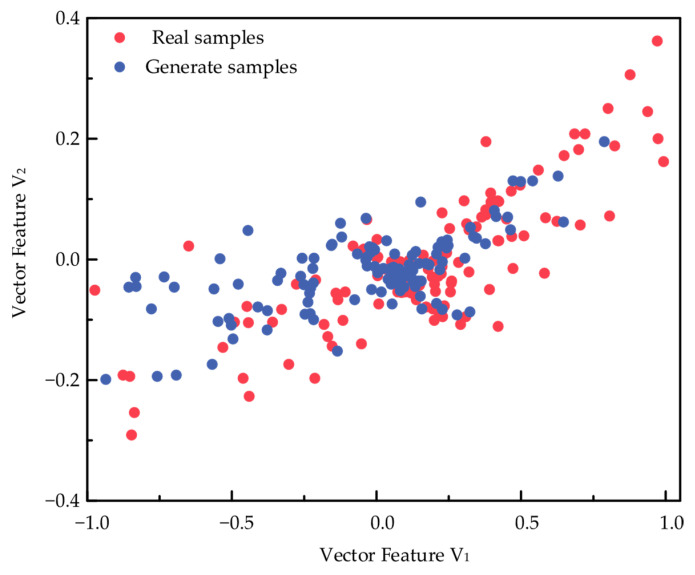
Demonstration of the distribution of generated samples and real samples.

To further reflect the similarity between the fault samples generated by the model generator used in this paper and the actual sample data distribution, the fault characteristics of the circuit breaker were ranked in importance by the previous three-stage XGBoost model, the actual fault samples and the generated fault samples were visualized by dimensionality reduction, and the data distribution of the generated samples and the real samples after dimensionality reduction and visualization is shown in Figure 11.

**Figure 12:**
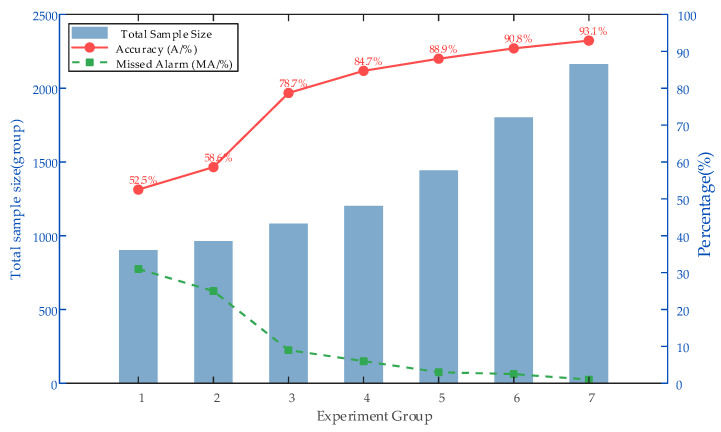
Trend of model performance under different sample ratios and sizes.

Artificial intelligence-based circuit breaker health assessment requires high-quality data, but there is a scarcity of fault samples in actual training. To simulate the real operation, the proportion and number of sample categories are continuously changed based on the proposed TCWGAN-GP in this paper to test the actual data enhancement effect, and the experimental results are shown in Table 9 and Figure 12.

From Table 9 and Figure 12, the results of Test 1 to Test 5 show that the more balanced the ratio of sample categories, the better the model performance. Meanwhile, from Test 5–Test 7, it can be seen that under the condition of balanced sample categories, the larger the number of samples, the better the model training performance. From the modeling results, it can be seen that the accuracy rate of Test 7 is 93.1%, which is 8.4% higher than that of Test 4; the missed alarm rate is 1.3%, which is 4.8% lower than that of Test 4. The performance of the model trained by data enhancement processing is better than that without data enhancement processing, which proves the effectiveness of the data enhancement method in this paper.

In order to verify the superiority of the TCWGAN-GP-based data generation method compared with other methods, this paper also compares TCWGAN-GP with the traditional data enhancement methods Random Oversampling (ROS) and Synthetic Minority Oversampling Technique (SMOTE), and puts the fault samples generated based on TCWGAN-GP, the fault samples generated based on ROS and the fault samples generated based on SMOTE into the three-stage XGBoost model as data sets for training, and the training results are shown in Table 10.

**Figure 13:**
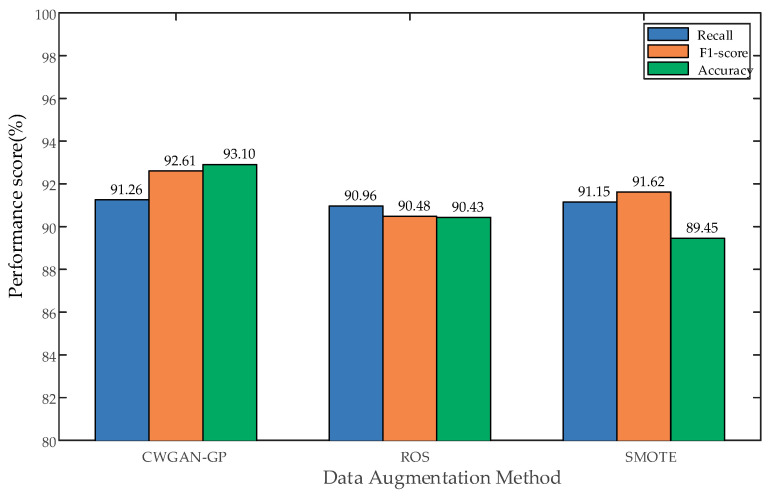
Performance comparison of different data enhancement methods (TCWGAN-GP vs. ROS vs. SMOTE).

From the results in Table 10 and Figure 13, it can be seen that the TCWGAN-GP-based data enhancement method is higher than the ROS and SMOTE-based data enhancement methods in the three evaluation metrics of Recall, F1-score, and Accuracy after the breaker data samples are fully balanced by different data enhancement methods. It shows that the TCWGAN-GP-based data enhancement method has the best training results in the three-stage XGBoost model proposed in this paper. It proves the superiority of the data enhancement method in this paper compared with the traditional data enhancement methods.

Furthermore, the proposed method focuses on learning general patterns of degradation rather than memorizing specific data distributions. The use of data augmentation via TCWGAN-GP also helps improve the robustness of the model under limited sample conditions. Nevertheless, it is acknowledged that real-world degradation processes may exhibit more complex and uncertain behaviors than those reproduced in laboratory experiments. Future work will involve validating the proposed approach using field data collected from operating substations to further assess its practical applicability.

## 5. Conclusions

In order to achieve accurate, real-time, and fine-grained condition assessment of substation circuit breakers, this paper proposes a circuit breaker health status assessment method based on TCWGAN-GP-based data augmentation and a three-stage XGBoost model, and proves the effectiveness of the proposed method through experimental validation, and obtains the following conclusions:

1. The three-stage XGBoost method for circuit breaker health state assessment is significantly better than the integrated model in terms of prediction effect, prediction time and other indicators, reducing fault discrimination time and saving arithmetic power.
2. The breaker fault sample data enhancement method based on the TCWGAN-GP model can enrich the data set while reducing the adverse effects of sample category imbalance on the model performance. Compared with the traditional data augmentation method, it improves the model prediction performance more significantly.
3. The paper achieves a refined assessment of line circuit breaker health status, fault type localization and fault degree determination, and provides an important reference for the fault repair schedule of the grid operation and maintenance personnel.

## Figures and Tables

**Table 1:** Health degree of equipment in distribution network.

Health Status	Colors	Definition
no defect (normal)	Green	Equipment in distribution network can perform functions as prescribed, each key parameter operation flexibility is high, and can operate reliably.
general defect	Blue	Equipment in distribution network can perform functions as prescribed, some parts have degraded performance, and risk resistance has decreased, but the impact is tiny.
significant defect	Yellow	Equipment in distribution network can perform functions according to regulations, the performance of some components is severely degraded, there are defects in risk tolerance, the impact is large.
emergency defect	Red	Equipment in distribution network cannot perform functions as prescribed, the impact is quite severe.

**Table 2:** Circuit breaker data characteristics.

Characteristic Categories	Characteristic Data	Dimension of Characteristics
Mechanical characteristics	Closing speed (*x*1) Closing time (*x*2) Breaking speed (*x*3) Breaking time (*x*4) Trip time (*x*5) Vibration signal (*x*6)	6
Electrical Characteristics	Breaking current (*x*7) Closing current (*x*8) Coil voltage (*x*9)	3
Insulation characteristics	SF6 gas pressure (*x*10)	1
Temperature Characteristics	Contact temperature (*x*11)	1
Environmental Characteristics	External temperature (*x*12) Air humidity (*x*13) Filthiness (*x*14)	3

**Table 3:** Fault simulation experiments.

Fault Types	Fault Simulation Modes	Sample Size
Normal	None	720 groups
Latch anomaly	Adding foreign objects	60 groups
Parting spring fatigue	Change spring pre-compression	60 groups
Closing spring fatigue	Change spring pre-compression	60 groups
Loose bolts in connection mechanism	Change the degree of bolt tightening	60 groups
Low coil voltage	Change the number of turns of the coil	60 groups
High contact temperature	Adjustment of current	60 groups
Insufficient SF6 gas pressure	Adjustment of SF6 gas pressure	60 groups
Buffer oil leakage	Change the amount of oil leakage	60 groups

**Table 4:** Detailed sample table of fault status.

Fault Type	General Faults	Critical Faults	Emergency Faults
Latch anomaly	20 groups	20 groups	20 groups
Parting spring fatigue	20 groups	20 groups	20 groups
Closing spring fatigue	20 groups	20 groups	20 groups
Loose bolts in connection mechanism	20 groups	20 groups	20 groups
Low coil voltage	20 groups	20 groups	20 groups
High contact temperature	20 groups	20 groups	20 groups
Insufficient SF6 gas pressure	20 groups	20 groups	20 groups
Buffer oil leakage	20 groups	20 groups	20 groups

**Table 5:** Algorithm performance comparison.

Model	Stage 1		Stage 2	Stage 3	Overall
	A/%	MA/%	A/%	A/%	A/%
XGBoost	98.5	1.3	96.8	97.6	93.1
SVM	96.6	3.4	95.1	96.3	88.5
GBDT	97.1	2.6	95.7	95.6	88.8
SAE	97.4	2.2	96.3	96.5	90.5
TabNet	97.9	1.8	96.2	96.8	91.2

**Table 6:** Performance comparison of different models by using single-stage assessment.

Indicators	XGBoost_Single	SVM_Single	GBDT_Single	SAE_Single	TabNet_Single
A/%	91.6	87.2	87.5	89.5	90.4
MA/%	3.6	5.4	4.9	4.6	3.8

**Table 7:** Time cost of single-stage mode.

Indicators	XGBoost_Single	SVM_Single	GBDT_Single	SAE_Single	TabNet_Single
Prediction time (s)	0.0241	0.240	0.262	0.310	0.385
Training time (min)	11	17	15	21	28

**Table 8:** Time cost of three-stage XGBoost model.

Indicators	Stage 1	Stage 2	Stage 3	Overall
Prediction time (s)	0.0041	0.0105	0.0025	0.0191
Training time (min)	2	5	8	15

**Table 9:** Data enhancement effect.

Test	1	2	3	4	5	6	7
Normal:Abnormal	12:3	12:4	12:6	12:8	12:12	15:15	18:18
Total sample size	900	960	1080	1200	1440	1800	2160
A/%	52.5	58.6	78.7	84.7	88.9	90.8	93.1
MA/%	30.8	25.4	9.5	6.1	3.2	2.4	1.3

**Table 10:** Comparison of different data augmentation methods (TCWGAN-GP vs. ROS vs. SMOTE).

Model	Data Not Fully Balanced			Data Fully Balanced		
	Recall	F1-Score	Accuracy	Recall	F1-Score	Accuracy
TCWGAN-GP	0.8214	0.8846	0.8500	0.9126	0.9261	0.9310
ROS	0.8284	0.8500	0.8591	0.9096	0.9048	0.9043
SMOTE	0.8167	0.8362	0.8550	0.9115	0.9162	0.8945

## Data Availability

The original contributions presented in this study are included in the article. Further inquiries can be directed to the corresponding author.
